# Case Report: Treatment of a Severe Puff Adder Snakebite Without Antivenom Administration

**DOI:** 10.4269/ajtmh.21-0291

**Published:** 2021-06-28

**Authors:** Masahiro Wakasugi, Toshiomi Kawagishi, Tomoya Hatano, Tadaki Shibuya, Hiroyuki Kuwano, Kotaro Matsui

**Affiliations:** 1 University of Toyama, Emergency & Disaster Medical Center, Toyama, Japan;; 2 Toyama Prefectural Central Hospital, Emergency Medical Center, Toyama, Japan

## Abstract

Antivenoms are the treatment of choice for managing lethal snakebites. However, antivenoms may not be available in instances where non-native vipers are kept in captivity. We report a case of a puff adder (*Bitis arietans*) bite treated without antivenom. A 23-year-old man was bitten on his left hand by a puff adder that he illegally kept in his house. The swelling spread rapidly to the upper arm and there was a risk of bleeding, suggesting the need for antivenom administration, but this could not be acquired because of lack of stock. We initiated fluid resuscitation and administered recombinant thrombomodulin (rTM) to prevent venom-induced consumption coagulopathy. In addition, hyperbaric oxygen (HBO) treatment was also performed to reduce local swelling. The patient recovered without complications after the multidisciplinary treatment. Further studies are needed to prove the safety and efficacy of rTM administration and HBO therapy as an adjunct or alternative therapy with antiserum for fatal snakebite.

Editorial office note: Figure 3 in this manuscript appears as Table 3 in the print version of this article.

## INTRODUCTION

Snakebite injuries are common life-threatening emergencies for the inhabitants of tropical Asia and Africa, mostly those involved in outdoor activities. The World Health Organization (WHO) predicted that the number of snakebite victims is likely to increase in the future because of climate warming and globalization.^1^

The puffadder (*Bitis arietans*) is the most common, highly venomous snake in Sub-Saharan Africa. ^2^ Puff adder bites can result in life-threatening injuries unless appropriate treatment, such as antivenom therapy, is provided. The incidences of puff adder bites outside Africa are rare but may result from snakes kept in captivity.^3^ This is concerning, as antivenom for the treatment of puff adder envenomation is not readily accessible in these regions. Puff adder venom contains a thrombolytic enzyme and causes tissue necrosis, hypotension, coagulopathy, thrombocytopenia, and spontaneous bleeding.^4^ We report our experience in treating a patient with a puff adder bite injury in the absence of an antivenom, using a combination of HBO therapy and rTM.

## CASE

**Figure 1.**
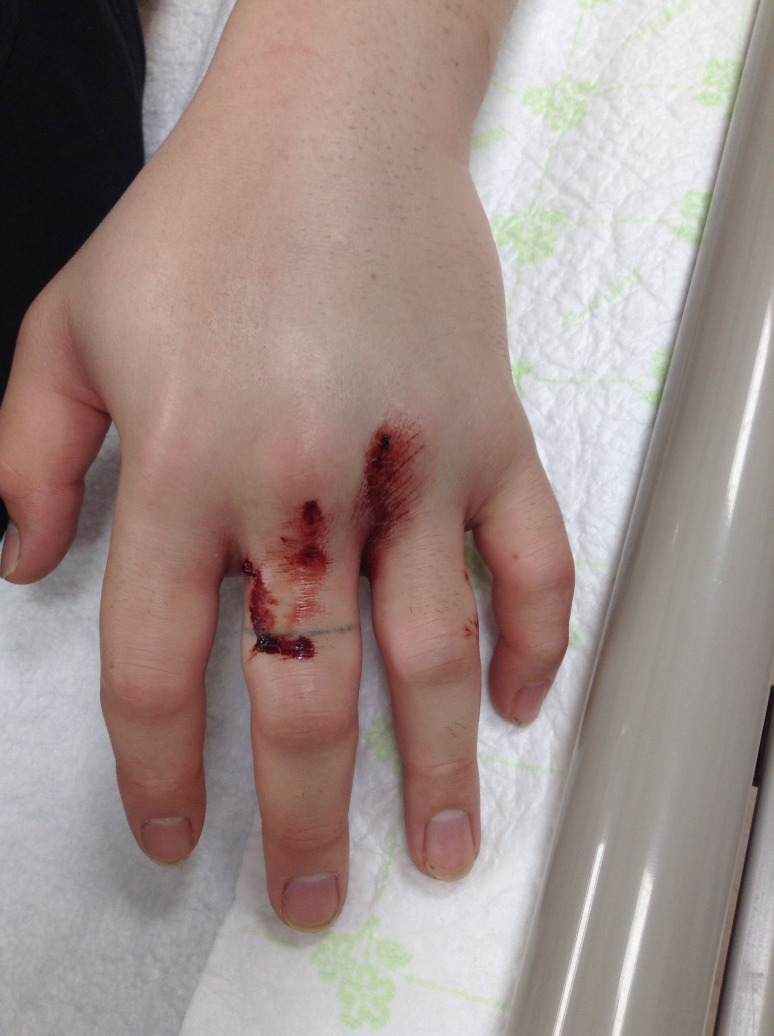
Swelling of hand on arrival (1 hour after bite). This figure appears in color at www.ajtmh.org.

A 23-year-old man came to the emergency room with complaints of swelling and severe pain in the left hand 30 min after being bitten by a puff adder that he illegally kept in his house. There were two puncture wounds approximately 1 cm apart on his left middle finger (Figure 1). There was no active bleeding from the wounds; however, marked swelling was observed on the dorsum of his hand, which was expanding to all his fingers. His vital signs were normal, and there was no neurological deficit.

**Figure 2.**
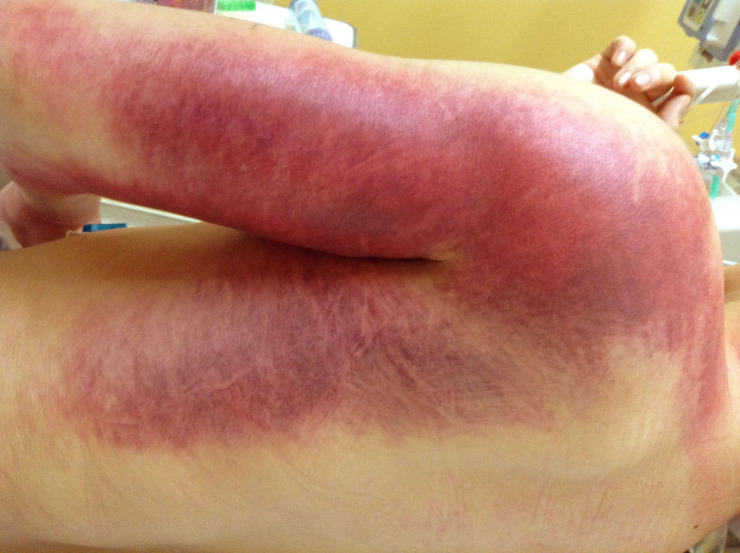
Progression of swelling to the back and subcutaneous bleeding (24 hours after bite). This figure appears in color at www.ajtmh.org.

**Figure 3:**
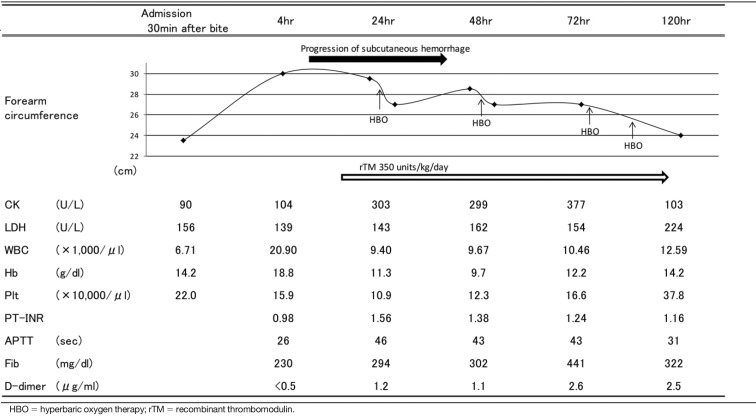
Clinical course

We initiated a saline drip and empirically administered an antibiotic (cefazolin, 1 g) and tetanus toxoid to the patient. Fentanyl (0.1 mg) was administered as required for pain relief. The swelling progressed rapidly to the forearm within the first hour. We considered administering antivenom, and we had inquired about the possibility of supplying antivenoms to zoological parks and research facilities in Japan; antivenoms are not available in Japan. His blood pressure tended to decrease gradually, and we then tried to maintain intravascular volume by increasing the amount of saline infusion (5,000 mL/day). The swelling progressed proximally to the elbow and armpit within 24 hr. In addition, the patient developed a marked subcutaneous hemorrhage in his arm to back (Figure 2). A full-body CT scan revealed no bleeding other than subcutaneous hemorrhage. Consequently, we inferred that there was a high probability that the patient would develop venom-induced consumption coagulopathy (VICC), and therefore decided to administer recombinant human soluble thrombomodulin, which is commonly used to treat disseminated intravascular coagulation (DIC) in Japan. We administered rTM at a dose of 350 units/kg for 5 days. After this, the hemoglobin levels of the patient increased to 18.8 g/dL after 4 hr (14.2 g/dL at the time of admission), then dropped to 9.7 g/dL, 48 hr after the bite. Twenty-four hr after the bite, his platelet count dropped from 220,000 to 100,000. His prothrombin time and international normalized ratio (PT-INR) increased from 0.98 to 1.56, and APTT from 26 s to 46 s. In addition, fibrinogen levels showed a mild elevation from 230 mg/dL to 294 mg/dL during this period. No blood transfusion or clotting factor replacement was performed during the course of the disease (Figure 3). Furthermore, coagulopathy peaked on the third day after admission and continued until the fifth day, while swelling worsened 72 hr after the bite. From the second to seventh day of injury, a total of six sessions of HBO therapy were performed by a monoplace chamber unit, each session extending for 90 min at 2 atm absolute (ATA) pressure. Immediately after HBO therapy, the forearm circumference decreased. Gradually, the forearm circumference returned—but the progression of swelling was controlled. The swelling peaked on day 4 and lasted for 14 days, but it did not cause compartment syndrome or tissue necrosis. There was no need for fasciotomy or surgical debridement. The patient did not develop hemolysis or acute kidney injury in the course of the disease and did not require hemodialysis. The patient was discharged on the 15th day after admission, with no complications other than mild contractures of the middle and ring fingers. Written informed consent was obtained from the patient for publication of this case report and any accompanying image.

## DISCUSSION

Poisoning from snakebite is a medical emergency that requires immediate attention. Antivenom is commonly used to treat lethal snakebite injuries. “Exotic” venomous snakes are seldom kept as pets; nevertheless, antivenom may not be available in regions outside their natural habitats. If antivenom cannot be administered after a lethal snakebite, as much symptomatic treatment as possible should be used to prevent possible complications.^5^ Puff adder (Bitis arietans) venom can cause serious systemic and local complications. Our patient presented with both symptoms. We were able to avoid adverse outcomes by using HBO therapy to alleviate local symptoms, and fluid resuscitation in addition to rTM administration to treat the systemic symptoms.

Schaeffer et al. reported that puff adder venom increases vascular permeability to proteins and red blood cells, primarily in the splanchnic regions, leading to hypovolemic shock and death.^6^ Our patient also experienced severe swelling of the upper arm from increased vascular permeability and required a large volume of fluid infusion to maintain circulation. Moreover, we performed HBO therapy to mitigate the risks of impaired blood flow and increased compartment pressure because of severe upper arm swelling. HBO therapy as adjunctive treatment has been reported to be effective in the management of snakebite injuries of the extremities.^7^^,^^8^ In this case, we managed to treat the patient without any complications or surgical procedures.

Puff adder venom is complex and has several components. One of the components is thrombin-like enzymes (TLEs),^9^ which consume fibrinogen and can in turn, lead to life-threatening hemorrhage.^10^

Thrombomodulin (TM) is a coenzyme that monitors thrombin production on the surface of vascular endothelial cell membranes, traps excess thrombin, and modulates the substrate specificity of thrombin from anticoagulant to antithrombotic. Recombinant thrombomodulin (rTM), developed in Japan for the treatment of DIC, promotes the activation of protein C by thrombin. The resulting activated protein C (APC) inhibits the production of thrombin by inactivating coagulation factors Va and VIIIa, thereby inhibiting the conversion of fibrinogen to fibrin.^11^ Therefore, we postulated that rTM would have an effect on VICC because of its action on thrombin. Ichiki et al. reported that the administration of rTM in addition to antivenom was effective against Yamakagashi snake (*Rhabdophis tigrinus*) envenomation, which causes life-threatening defibrinating coagulopathy.^12^ In our case, there was a decrease in platelet count and mild coagulopathy. However, there was no decrease in fibrinogen levels. This suggests that the inhibition of thrombin generation by rTM may have suppressed fibrinogen consumption by TLEs. In this case, the effect of early administration of TM may have resulted in higher fibrinogen from the inflammatory response without a decrease in fibrinogen. Because the consumption of other coagulation factors cannot be suppressed by rTM, if the coagulopathy becomes severe, fresh-frozen plasma is one of the treatment options;^9^ but fortunately in this case, no severe coagulopathy occurred that required blood transfusion.

In conclusion, we reported a patient with a severe envenomation by puff adder snakebite for which antivenom was not available. A multidisciplinary treatment approach using rTM and HBO therapy resulted in the discharge of the patient with no complications. Of course, treatment with antivenom is the gold standard for fatal snakebites, but we also need to be prepared for when antivenom is not possible. The effect of puff adder venom in this case was mainly because of increased vascular permeability, and coagulopathy was mild and thought to be consumption coagulopathy by subcutaneous bleeding—not typical symptoms of VICC. It is difficult to assess whether the lack of VICC was because of the effect of rTM. Further studies are needed to demonstrate the safety and efficacy of rTM in treating VICC caused by fatal snakebites.
